# Pomegranate Juices: Analytical and Bio-Toxicological Comparison of Pasteurization and High-Pressure Processing in the Development of Healthy Products

**DOI:** 10.3390/foods14020315

**Published:** 2025-01-18

**Authors:** Francesco Cairone, Stefania Cesa, Irene Arpante, Simonetta Cristina Di Simone, Alejandro Han Mendez, Claudio Ferrante, Luigi Menghini, Antonello Filippi, Caterina Fraschetti, Gokhan Zengin, Simone Carradori, Marialucia Gallorini, Luisa Mannina, Mattia Spano

**Affiliations:** 1 Department of Chemistry and Technologies of Drug, “Sapienza” University of Rome, 00185 Rome, Italy; francesco.cairone@uniroma1.it (F.C.); irene.arpante@uniroma1.it (I.A.); alejandrohan.mendez@uniroma1.it (A.H.M.); antonello.filippi@uniroma1.it (A.F.); caterina.fraschetti@uniroma1.it (C.F.); luisa.mannina@uniroma1.it (L.M.); mattia.spano@uniroma1.it (M.S.); 2 Department of Pharmacy, University “G. d’Annunzio” of Chieti-Pescara, 66100 Chieti, Italy; simonetta.disimone@unich.it (S.C.D.S.); claudio.ferrante@unich.it (C.F.); luigi.menghini@unich.it (L.M.); simone.carradori@unich.it (S.C.); marialucia.gallorini@unich.it (M.G.); 3 Department of Biology, Science Faculty, Selcuk University, Konya 42130, Turkey; gokhanzengin@selcuk.edu.tr

**Keywords:** pomegranate juice, nutritional value, HPLC-DAD, ^1^H-NMR, vitamin C, antioxidant, toxicological analyses, enzyme inhibition, HS-SPME-GC-MS

## Abstract

Two different produced and packaged commercial typologies of pomegranate juice were analyzed for their physicochemical, nutritional, and biological properties. The effects of classical pasteurization (PJ) and high-pressure processing (HP), applied during the productive cycle, were evaluated through several advanced analytical methods, such as CIEL*a*b* colorimetry, HPLC-DAD, DI-ESI-MS and MS/MS, and NMR analyses. Moreover, the exerted biological activity of the two pomegranate juices was monitored through Total Phenolic and Total Flavonoid Contents, antiradical, antioxidant and chelating activity. The potential inhibition of key enzymes of degenerative processes (cholinesterases, tyrosinase) and diabetes (amylase, glucosidase), the allelopathy toward *Cichorium intybus*, *Dicondra repens*, and *Diplotaxis tenuifolia*, and the in vivo toxicity on brine shrimp were also evaluated. The two different applied processing techniques analyzed impacted the bioactive compound’s preservation differently, modifying the phytocomplex profile. HP significantly degrades punicalins and punicalagins, better preserving anthocyanins, if compared to PJ’s impact. Sensory qualities, antioxidant activity, enzymatic inhibition, and ecotoxicological potential were differently impacted by the two applied processes. The obtained results can be beneficial for finding the optimal processing conditions that balance microbial safety with nutritional value preservation, contributing to the development of healthy pomegranate juice products.

## 1. Introduction

Fruits of the pomegranate (*Punica granatum* L.) and other parts of this plant have long been thought to be beneficial to human health. Fresh fruits as well as fruit juices have recently been elevated by nutritionists to the status of functional foods, recognizing superiority over other fruits and a special position in terms of wide healthy applications [1]. Their recognized added value as anti-radical and antioxidant aliments is strictly associated with an exceptional ellagitannin phytocomplex content which exerts a potent anti-inflammatory action. These compounds, along with other polyphenols, like anthocyanins (mainly delphinidin, pelargonidin and cyanidin glycosides), contribute to the fruit’s therapeutic potential in preventing and managing chronic diseases, including cardiovascular, inflammatory, and metabolic disorders [1,2].

Beyond its bioactive components, pomegranate is rich in essential nutrients, such as sugars (glucose and fructose), organic acids (oxalic and tartaric acids), vitamins, minerals, and fatty acids like punicic acid [3]. Transformed products deriving from fruits, and other separated botanical parts of *P. granatum*, are largely studied for the prevention and management of inflammation-triggered chronic diseases, diabetes, or carcinogenesis [4]. The benefits of pomegranate are primarily attributed to the synergistic interaction among its polyphenolic compounds and vitamin C, amplifying the protective effects beyond those of individual components [5]. The protective potential from chronic diseases of the phytocomplex is under evaluation in dozens of studies focused on its anti-radical and antioxidant activity, cardioprotective and antimicrobial effects, and anti-inflammatory and antiproliferative potential [6,7,8,9,10,11].

Given the widespread consumption of pomegranate as a juice product, preservation of its bioactive properties during processing is of paramount importance to obtain final products that still retain the original nutritional value. Compromises need to be made between shelf-life duration, growth control of microbes, and product quality [12,13].

Several studies showed the effect of various applied techniques and thermal treatments on the degradation/transformation of bioactive compounds, with the aim to indicate the best processing procedures [14,15,16]. Traditional thermal pasteurization methods, such as high-temperature short-time (HTST) processing, are commonly employed to ensure microbial safety and extend shelf life. However, these methods often compromise sensitive phytochemicals, such as anthocyanins and vitamin C, leading to the degradation of some bioactive compounds and undesirable changes in sensory and nutritional qualities, such as color, taste and aroma, texture, and appearance [17,18,19]. In response, non-thermal technologies, including high-pressure processing (HPP), ultraviolet (UV) pasteurization, and pulsed light (PL) treatment, have gained significant attention for their ability to inactivate microorganisms while minimizing the impact on bioactive compounds and sensory attributes. For instance, HPP has shown efficacy in preserving anthocyanins, color, and antioxidant activity. However, it needs to be refrigerated and has a shorter shelf life compared to thermally treated juices [20,21,22]. Similarly, UV and PL treatments have been shown to have microbial inactivation potential associated with lower thermal damage, making them promising alternatives for preserving high-quality juices [23]. Among the so-called non-thermal technologies applied to food, high-pressure processing is acquiring increasing interest. This is considered an innovative method to extend food shelf life without resorting to classic pasteurization, and its effects have been previously studied on blueberry and mulberry juices [24,25].

In relation to these properties, the present study aims at evaluating and comparing the physicochemical, nutritional, and biological properties of two different pomegranate juices obtained by traditional fruit squeezing and pasteurization (PJ) or by using cold water for high-pressure and ultrafiltration processing (HP). The focus is on understanding how these two different industrial processing techniques impact the preservation of bioactive compounds, antioxidant activity, and sensory qualities, alongside their potential enzyme inhibition and ecotoxicological and allelopathic effects. By integrating different advanced analytical methods such as colorimetry, HPLC-DAD, ^1^H NMR, HS-SPME-GC-MS, and bioassays, this research seeks to identify and guide the optimal processing conditions which guarantee microbial safety, nutritional value, and sustainability, contributing to the development of less impactive, high-quality functional food products from pomegranate fruits.

## 2. Materials and Methods

Ethanol, methanol, and acetonitrile (HPLC-grade) and other reagents were obtained from Merck Science Life srl (Milan, Italy). Bidistilled water was obtained from Carlo Erba (Milan, Italy).

The two samples, pasteurized pomegranate juice (PJ) obtained by traditional fruit squeezing and pasteurization and the high-pressure processing juice (HP) obtained using cold water at high pressure and ultrafiltration, were kindly provided by “Masseria Fruttirossi srl.” (Contrada Terzo Dieci snc, 74011, Castellaneta, Taranto, Italy) in February 2024 according to their industrial processing operations. The production process is completely integrated into the company, which directly manages all aspects related to farming, harvesting, and processing, according to good agricultural practice.

### 2.1. Sample Preparation

The two typologies of 100% pomegranate juices, produced and packaged for normal distribution on market, were immediately used or stored, according to the instructions on the label (PJ at room temperature and HP at temperature of 4 ± 2 °C) and used as such or after freeze-drying. All analyses were performed within the expiring dates (PJ: stored at room temperature and to be consumed preferably before 23 July 2025; HP: stored in the fridge at maximum 6 °C and to be consumed before 30 May 2024).

CIEL*a*b* and HPLC-DAD analyses were carried out on fresh juices, whereas DI-ESI-MS, MS/MS, NMR, TPC, TFC, DPPH, ABTS, CUPRAC, enzyme inhibition, and in vivo toxicity analyses were carried out starting from freeze-dried samples. In particular, juice aliquots were freeze-dried at −55 °C and 0.5 mbar using a BUCHI Lyovapor L-200 (BUCHI Italia s.r.l., Cornaredo, Italy) and stored at −20 °C until analysis.

### 2.2. CIEL*a*b* Analysis

The CIEL*a*b* parameters (L*, a*, b*, C*_ab_, and h_ab_) were determined, using a MetaVue X-rite TM colorimeter, (Grand Rapids, MI, USA) featuring an LED illuminant/45–0° and Color iQC10 software (Grand Rapids, MI, USA), on fresh pomegranate juice samples, stored according to the label and analyzed before the expiration date (HP) or the recommended date of consume (PJ). The analyses were performed on the samples as follows: apparent color in the presence of turbidity, after filtration (PJ_1_, HP_1_) and/or double filtration (PJ_2_, HP_2_) on paper (color in the absence of turbidity), and after concentration (PJ_3_, HP_3_) by volume reduction (10:1). CIEL*a*b* parameters enabled us to characterize the chromatic properties of the different samples [26]. Overall color differences were calculated as reported in Supplementary Materials (Equation (S1)).

### 2.3. HPLC-DAD Analysis

Fresh juices (PJ and HP) were filtered using Whatman^®^ grade 1 filter paper (Merck, Milan, Italy) followed by further filtration with a Millex^®^—LG filter (Low Protein Binding Hydrophilic PTFE 0.22 µM membrane) (Merck Science Life S.r.l., Milan, Italy). A volume of 100 µL of undiluted juice was injected into an HPLC-DAD system (Perkin Elmer, Milan, Italy) equipped with a Series 200 LC pump, a Series 200 DAD, and a Series 200 autosampler, using TotalChrom 6.3.2 Perkin Elmer software for plotting data. Analyses were conducted following the method described by Fraschetti et al. [13], with slight modifications. The system employed a Nucleosil RP-18 column (5 µm) with a linear gradient of acetonitrile and acidified water (5% acetic acid, *v*/*v*), transitioning from 100% to 80% aqueous phase over 40 min at a flow rate of 1 mL/min. Detection was performed at 360 nm for ellagitannins and 520 nm for anthocyanins. Calibration curves were prepared for punicalin (α + β; RT = 5.0–5.2, y = 8.48x + 50.03; R^2^ = 0.9976), punicalagin (α, RT = 6.9; β, RT = 9.7; y = 3.70x + 2.70; R^2^ = 0.9999), ellagic acid (RT = 30.4; y = 1.16x − 38.34; R^2^ = 0.9995), and cyanidin-3-glucoside (RT = 28.3; y = 1.66x − 77.13; R^2^ = 0.9994), which were quantified in mg/mL of juice.

The analysis of vitamin C was performed using an Agilent 1200 SL Rapid Resolution Liquid Chromatograph with an autosampler and an 80 Hz diode array detector (Agilent Technologies Inc., Santa Rosa, CA, USA). A ZORBAX Eclipse Plus C-18 column (5 µm, 4.6 × 250 mm) was used with a Flexar UV/VIS detector in isocratic mode (90% 25 mM NaH_2_PO_4_, pH 2.5, and 10% methanol) at a flow rate of 0.8 mL/min, with detection at 270 nm. The calibration curve for vitamin C (y = 3.10 × 10^−5^x − 1.35; R^2^ = 0.9964; RT = 3.70 min) was used to quantify its content, expressed in mg/kg.

### 2.4. DI-ESI-MS and MS/MS Analyses

The freeze-dried HP and PJ samples were dissolved in methanol to obtain clear 7 mg/mL solutions, which were infused at 10 μL min^−1^ in the ESI source of a LTQ XL linear ion trap (Thermo Fischer Scientific, Waltham, MA, USA). The source parameters were set as follows (values used for negative mode in the brackets): source voltage = 5.0 (4.50) kV; capillary voltage = 40 (−30) V; capillary temperature = 275 °C; tube lens voltage 80 (−60) V, sheath gas and sweep gas flow rates, 4 and 5, respectively (arbitrary units). Each spectrum, acquired over the 100–2000 *m*/*z* range, arose from averaging 10 full scans, each one consisting of 5 micro scans. A targeted MS/MS measurement in both positive and negative polarity modes was performed, isolating all the analytes whose *m*/*z* ratio agreed with the values predicted for anthocyanins and their derivatives. The precursor ion isolation width was 1–2 Da and the normalized collision energy was set to the value needed to reduce the intensity of the precursor ion to about 10%. The MS-tandem spectra were compared with those collected in the literature [27,28].

### 2.5. NMR Analysis

Samples obtained by freeze-drying 10 mL juices were solubilized in 4 mL of 100 mM phosphate buffer/D_2_O, containing TSP (3-(trimethylsilyl) propionic acid sodium salt) 1 mM as internal standard. Mono-dimensional ^1^H and two-dimensional ^1^H–^1^H TOCSY NMR experiments were carried out using a 600 MHz spectrometer (JNM-ECZ-600R (JEOL Ltd., Tokyo, Japan) ( equipped with a 5 mm FG/RO DIGITAL AUTOTUNE probe, applying the same experimental parameters previously described [29].

JEOL Delta software (v5.3.1) was used to manually phase and base correct the spectra and to integrate the signals used for the quantification of metabolites. Concentrations of metabolites were measured with respect to the internal standard TSP. For each sample, three replicates were carried out and the results were expressed as mg/mL of fresh juice ± SD.

### 2.6. Total Phenolic Content and Total Flavonoid Content

The Total Phenolic Content (TPC) was measured using the Folin–Ciocâlteu reagent and the Total Flavonoid Content (TFC) was measured using aluminum chloride under basic conditions, as previously detailed [1]. The obtained solutions of the freeze-dried samples PJ and HP with the reagents were incubated in the dark for 30 min at room temperature. Relative absorbances were read, respectively, at 760 for TPC and at 430 nm for TFC, using a JASCO UV-VIS spectrophotometer. A reference curve was prepared with different concentrations of gallic acid (R^2^ = 0.9991), and TPC data were expressed as mg GAE/g freeze dried material; additionally, a reference curve was prepared with different concentrations of rutin (R^2^ = 0.9989), and TFC data were expressed as rutin equivalents.

### 2.7. Antiradical, Antioxidant, and Chelating Activity

The radical scavenging activity on the 1,1-diphenyl-2-picrylhydrazyl (DPPH) radical and on the 2,2′-azino-bis(3-ethylbenzothiazoline)-6-sulphonic acid (ABTS) radical cation was tested according to Balli et al. [1]. The freeze-dried samples were treated with appropriate solutions of DPPH or ABTS (preincubated in potassium persulphate for 12–16 h) and incubated in the dark for 30 min at room temperature. Absorbance values were read, respectively, at 517 nm for DPPH assay and at 734 nm. Reference curves were prepared with different concentrations of Trolox (R^2^ = 0.9990), and the radical scavenging activity of the samples was expressed as Trolox equivalents.

The cupric ion reducing activity (CUPRAC) on CuCl_2_ in the presence of neocuproin in ammonium acetate buffer, the ferric reducing antioxidant power (FRAP, Fe^3+^ and 2,4,6-tris(2-pyridyl)-*s*-triazine), and the total antioxidant capacity by the phosphomolybdenum (PBD) assay (sulphuric acid, sodium phosphate and ammonium molybdate) were measured according to Balli et al. [1]. As previously described in detail, the freeze-dried samples were treated with appropriate volumes of opportunely prepared solutions of reagents (CUPRAC and FRAP), incubated at room temperature in the dark for 30 min, and the absorbance values were read, respectively, at 450 nm (CUPRAC) and at 593 nm (FRAP). The solutions obtained with PBD reagents were incubated in the dark for 90 min at 95 °C and the absorbance values read at 695 nm. In all three assays, the results were reported as Trolox equivalents. Metal Chelating Activity on ferrous ions (EDTA assay) was evaluated on FeCl_2_ and ferrozine solutions, according to Locatelli et al. [30]. The freeze-dried samples, treated with appropriate volumes of opportunely prepared solutions of reagents, were incubated for 10 min at room temperature and read at 562 nm. Ethylenediaminetetraacetic acid (EDTA) was used as reference standard and the chelating activity was expressed as EDTA equivalents.

### 2.8. Enzyme Inhibition

The enzyme inhibition towards acetylcholinesterase (AChE, electric eel, Type-VI-S, EC 3.1.1.7), butyrylcholinesterase (BChE, horse serum, EC 3.1.1.8)), tyrosinase (mushroom, EC 1.14.18.1), amylase (porcine pancreas, EC 3.2.1.1), and glucosidase (*Saccharomyces cerevisiae*, EC 3.2.1.20) was determined on the freeze-dried samples according to Balli et al. [1]. Standard inhibitors were used as positive controls (galantamine for cholinesterases; kojic acid for tyrosinase; and acarbose for amylase and glucosidase) and the inhibitory activities were expressed as corresponding equivalents, as previously described.

### 2.9. In Vivo Toxicity

#### 2.9.1. Preparation of Test Sample Concentration

Stock solutions of *Punica granatum* HP and PJ juices were prepared at a concentration of 100 mg/mL using water as the solvent and further diluted to obtain the desired concentration range for testing. For the allelopathy assay, distilled water was used for the dilution, with a concentration range from 2.5 to 40 mg/mL, while artificial sea water was used for the brine shrimp lethality assay, with a concentration range from 0.078 to 10 mg/mL.

#### 2.9.2. Allelopathy Assay

A Petri dish-based experiment was carried out to evaluate the potential phytotoxic activity of *P. granatum* juices in a concentration range from 40 to 2.5 mg/mL. Commercial seeds of *Cichorium intybus*, *Dicondra repens*, and *Diplotaxis tenuifolia* were selected for the test due to their fast germination rate and sensitivity. The producers guarantee a germination rate of the seeds >70%, also confirmed by preliminary germination tests. The assay was conducted in 90 mm Petri dishes, each containing a double-layered filter paper disk, previously soaked with 3 mL of the plant extract at different concentrations. Distilled water was used as negative control. The seeds were surface-sterilized using a diluted (NaClO:dH_2_O, 1:9) commercial bleaching liquid for 10 min. After thoroughly rinsing the seeds with sterile distilled water to remove any traces of bleach, 10 seeds of the corresponding seed variety were arranged on the filter paper disks, previously divided into 3 distinct areas. Petri dishes were then sealed with parafilm to ensure a closed-system model and incubated in darkness at room temperature (25 ± 2 °C) for 96 h. The criterion for determining germination was the emergence of a radicle at least 2 mm in length, exhibiting typical geotropic curvature. Seeds that only swelled but did not fully germinate were excluded. Radicle length was measured and categorized by elongation rate: low (<0.4 cm), medium (0.5–0.9 cm), or high (>1 cm). The number of germinated seeds and their radicle lengths were recorded after 96 h, and the results were reported as mean germination percentage and growth percentage, which is the mean of seedlings length compared to control samples.

#### 2.9.3. Brine Shrimp Toxicity Bioassay

The toxicity assessment was carried out as described by Meyer et al. [31] and McLaughlin et al. [32] on freshly hatched brine shrimps (nauplii). Triplicate samples of each concentration were tested in a range of concentrations between 0.078 and 10 mg/mL in glass tubes containing 5 mL of artificial sea water and 10 nauplii each. Surviving brine shrimp nauplii were counted after 24 h and the median lethal concentration (LC_50_) was calculated using GraphPad Prism 8.0.2 software. For the acceptability of the test, up to 10% of mortality in the control was admitted.

### 2.10. Statistical Analysis

One-way ANOVA, followed by Tukey’s multiple comparisons test, was applied to underline significant differences among the considered samples. GraphPad Prism 8.0.2 software was used for this purpose.

## 3. Results and Discussion

### 3.1. Color CIEL*a*b*Analysis

**Figure 1:**
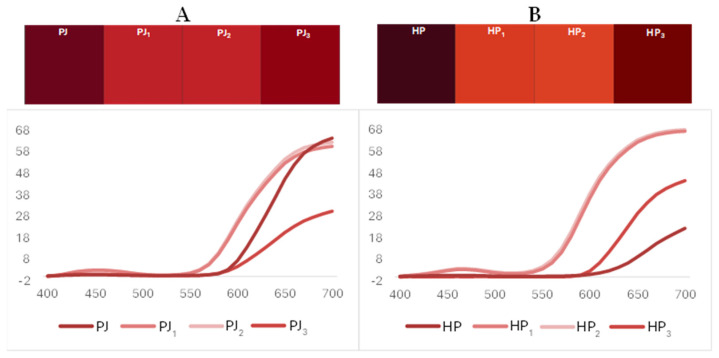
Palette and reflectance curves of turbid, filtered and concentrated PJ (Panel **A**) and HP (Panel **B**) juices. Non-reported RSD % values are within 1–2%.

To obtain information about their chromatic properties, the two raw juice samples were subjected to a CIEL*a*b* color analysis after simple transformation processes. The color of food has long been considered a key quality indicator [33]. In particular, the color of pomegranate juices has been used to assess their physicochemical properties, as well as the effects of technological processes and storage conditions [34,35]. Real color and apparent color were evaluated by filtering the samples onto paper until they appeared clear to the naked eye. Two consecutive filtration processes (PJ_2_ and HP_2_) were considered sufficient to obtain clear solutions. Moreover, concentration (PJ_3_, HP_3_) under vacuum enabled us to better evaluate differences in terms of reflectance curves and CIEL*a*b* parameters. The palettes and reflectance curves of the two analyzed samples are shown in Figure 1. Both raw samples exhibited a high turbidity, shown by the palettes and the comparison between the reflectance curves, which can be due to the presence of undissolved polyphenols or partially insoluble fibers. These were probably co-extracted from the fruits into the juice during their preparation. The presence of undissolved polyphenols or fibers does not represent a problem for the juices; on the contrary, it could be representative of healthy biomolecules. Anyway, it confers turbidity to the samples, whose pigment’s color remains only partially expressed. The turbidity was almost completely removed during the first filtration, as shown by the palettes and the associated reflectance curves. The relative CIEL*a*b* parameters are reported in Table 1. A residual minimal difference between samples filtered once or twice was shown by the ΔE (overall color difference) values (PJ_2_ vs. PJ_1_, 1.83; HP_2_ vs. HP_1_, 2.03), so that the aim could be considered reached with only one filtration step. Concentrated samples were then obtained using these once-filtered samples as starting materials. By the analysis of concentrated juices, we can enhance the chromatic character of the pigments, while avoiding the presence of sediments which compress the reflectance curves and relative color parameters downwards. This is only partially shown by the comparison of color parameters, because the two samples undergo different behavior. Concentrated PJ shows color parameters more similar to the filtered one, and the HP is more similar to the raw sample. This behavior, as well as the evident differences in the expressed color (ΔE = 14.66, filtered PJ vs. filtered HP), preluded a quite different composition of pigment between the two processed juices, which was confirmed by the HPLC analysis.

### 3.2. Quali-Quantitative HPLC Analysis

**Figure 2:**
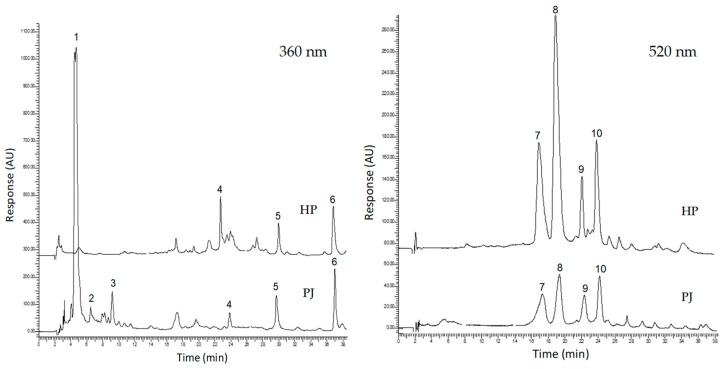
HPLC-DAD profiles of HP and PJ juices at 360 and 520 nm. (1) Punicalin (α + β), (2) α-punicalagin, (3) β-punicalagin, (4) ellagic acid derivative, (5) ellagic acid glucoside, (6) ellagic acid, (7) del-3,5-diglucoside, (8) cya-3,5-diglucoside, (9) del-3-glucoside, (10) cya-3-glucoside.

PJ and HP juices, prepared for HPLC analysis as previously described, were submitted to quali-quantitative analysis, performed at 270 nm for the analysis of vitamin C, at 360 nm for the ellagitannin profile, and at 520 nm for the anthocyanin profile. Regarding vitamin C content, HPLC-DAD analysis revealed a notably higher concentration in PJ (284 mg/kg) compared to HP (185 mg/kg). This difference is due to the lower thermal stress in HP, where enzymatic degradation of vitamin C can still occur due to the longer processing time under pressure. In contrast, the rapid heating and subsequent cooling in thermal pasteurization might reduce degradation by inactivating ascorbate oxidase more effectively [36,37]. The analysis of PJ at 360 nm made it possible to identify and quantify the peaks of α- and β-punicalin (two collapsing peaks at T_r_ = 5.0–5.2), α- and β-punicalagin (T_r_ = 6.9 and 9.7, respectively), an ellagic acid derivative (T_r_ = 23.7), an ellagic acid glucoside (T_r_ = 29.9), both quantified as µg/mL equivalents of ellagic acid, and ellagic acid (T_r_ = 37.1). Chromatograms are reported in Figure 2 and quantitative data are shown in Table 2. The peak assignment of punicalins and punicalagins, as well as of ellagic acid, was performed using reference standards (Supplementary Materials, Table S1, Figures S1 and S2), while for ellagic acid derivative and ellagic acid glucoside, a comparison with the literature was necessary. Specifically, as reported by Esposto et al. [38] and Gómez-Caravaca et al. [39], ellagic acid glucoside is one of the most represented ellagic acid derivatives in pomegranate juices. The significant peak at a T_r_ of 23.7 min in HP juice appears to be compatible with one of the different ellagic acid derivatives reported in the literature [12]. As it cannot be definitively attributed to a specific one, it was identified as an ellagic acid derivative.

Contrary to data from the literature [12,40] and previous research [1,13], the most represented ellagitannins in the PJ sample were punicalins, totaling about 400 mg/L juice (Table 2). A similar result has already been reported in the literature by Esposto et al. [38], who found high levels of punicalins by the analysis of commercial pomegranate juices. Punicalins and punicalagins are completely lost during HP processing. Moreover, ellagic acid is differently distributed in its derivatives but comparably represented (73–77 mg/L as sum) in PJ and HP. The higher content of free ellagic acid in HP (about 34 mg/L) may be due to the degradation of ellagitannins (punicalins and punicalagins) during the processing. As reported in the literature, only six anthocyanin pigments, represented by the 3-mono and 3,5-diglucosylated anthocyanidins, cyanidin, delphinidin and pelargonidin, are found in pomegranate juices [41,42]. According to the experiment’s results, the four derivatives of delphinidin and cyanidin were well represented, and small, unquantifiable amounts of mono and di-glycosylated pelargonidin were found. In contrast to the ellagitannins, the red pigments of the anthocyanins were more present in HP than in PJ (about 130 vs. 76 mg/L, expressed as a sum in Cya-3-glucoside equivalents). Nevertheless, Del-3,5-diglucoside and, furthermore, Cya-3,5-diglucoside are significantly more represented in HP than in PJ, while Del-3-glucoside is comparable and Cya-3-glucoside is only slightly more represented.

The results suggest that high-pressure technology more effectively preserves anthocyanins compared to pasteurization but significantly degrades punicalins and punicalagins, only partially transformed in free ellagic acid which is more represented in HP. This finding aligns with Cairone et al. [28], who reported that high-pressure processing better preserved anthocyanins in Clery strawberries compared to conventional methods, emphasizing the technology’s potential in maintaining polyphenolic compounds during fruit processing. Moreover, pasteurization appears to be more effective in preserving vitamin C, inactivating enzymes involved in its degradation.

### 3.3. MS Based Anthocyanin Profiling

**Figure 3:**
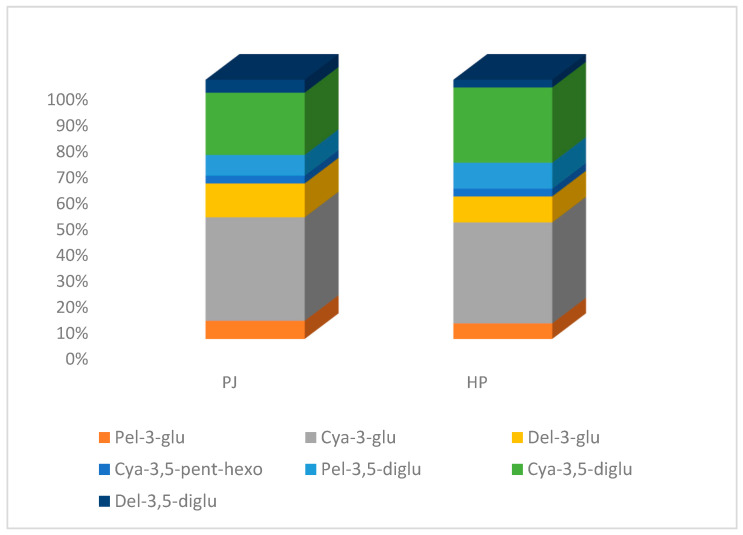
Intensity percentages of the individual anthocyanins in the PJ and HP juice samples.

The anthocyanins’ profiling pointed out by the HPLC-DAD analysis was substantially confirmed by the DI-ESI-MS results. The identification of seven anthocyanins, four of which were already identified by HPLC-DAD analysis, was obtained by comparing the MS-tandem spectra with those reported in the literature and schematized in Table 3. Furthermore, the remarkable sensitivity of the ESI–mass spectrometry enabled the detection of a small amount of Cya-3,5-pentoside-hexoside, identified for the first time in pomegranate juice by Fischer et al. [12]. Figure 3 reports the percentages by intensities of the individual anthocyanin in both samples, pointing to a larger relative abundance of mono- and diglycosylated derivatives of cyanidin, which represent about 65–70% of the overall anthocyanin fraction, partially confirming data from the HPLC analysis. The ESI mass spectrum measured in negative polarity is dominated by the presence of citric acid and its fragment, as confirmed by the NMR results.

### 3.4. NMR-Based Metabolite Profiling

Metabolite identification was carried out using information obtained by ^1^H signals (chemical shift, multiplicity, J coupling constants), ^1^H-^1^H TOCSY spin–spin correlations, and literature data regarding NMR analysis of pomegranate [43,44]. The applied NMR approach allowed us to obtain a comprehensive picture of the most abundant water-soluble metabolites in the analyzed juices. Eleven amino acids and derivatives (alanine, asparagine, aspartate, GABA, glutamine, isoleucine, leucine, phenylalanine, threonine, tyrosine and valine), six sugars and polyols (glucose, fructose, galactose, sucrose, maltose and myo-inositol), four organic acids (citric, formic, fumaric and gallic acids), ethanol, uridine, and trigonelline were identified and are quantified in Table 4.

From a qualitative point of view, both samples presented the same metabolites, although from a quantitative point of view, pasteurized juice was characterized by the highest amount of all the compounds, except for gallic acid.

Sugars were the most abundant group, mainly represented by glucose, followed by fructose. It is noteworthy to underline that in the samples analyzed herein, the glucose content is 3–5 higher with respect to fructose, which is different from data in the literature, which has reported similar amounts of both sugars in this matrix [4,41]. Among organic acids, citrate, responsible for the sour taste and antioxidant activity, was the most abundant, with more than double the amount in the pasteurized sample. The amino acids profile observed herein was qualitatively the same with respect to profiles previously reported by other NMR studies, with glutamine, GABA, and aspartate being the most abundant.

### 3.5. Total Phenolic and Total Flavonoid Content, Antioxidant Properties, and Anti-Radical Potential of the Pomegranate Juices

The TPC and TFC, as well as all antioxidant, antiradical, and enzyme inhibitory potential tests of the two juices, were measured on lyophilized samples (Table 5) [45]. The TPC of PJ was 15.94 mg GAE/g, and the HP juice showed a lower value of 11.47; in a similar way, the TPC of PJ was higher (4.99) than the value (1.93) found in HP. These results substantially confirm the results of the HPLC analysis, by which a higher content of hydrolysable tannins, gallagic compounds, ellagic compounds, and vitamin C was found in pasteurized pomegranate juice, and a lower content was found in the high-pressure-treated juice (the PJ values were greater than HP by approximately 1.4 times for TPC and 2.6 times for TFC, and flavonoids were not specifically found in the two analyzed samples). Similarly, all the tests performed for the evaluation of the antioxidant, anti-radical, and chelating properties (DPPH, ABTS, CUPRAC, and FRAP between 1.6 and 1.8 times greater for PJ than HP; EDTA 2.6 times greater; and PBD 1.2 times greater) showed an antioxidant capacity and an anti-radical potential for PJ that was about 2-fold higher with respect to HP (1.2-fold higher for PBD; values ranging between 1.6- and 1.8-fold higher for DPPH, ABTS, CUPRAC and FRAP; and 2.6-fold higher for EDTA) [1].

### 3.6. Enzymatic Inhibition

Given the peculiar phytochemical profile analyzed so far, both pomegranate juices were furthermore tested for their ability to inhibit the activity of key enzymes involved in physiopathological processes in humans (Table 6), as also demonstrated for other pomegranate juice-based products (dried pomegranate leaves, juice concentrate, juice powder, or concentrated solution) [46,47,48,49,50].

Inhibitory data against cholinesterases (AChE and BChE), amylase and glucosidase were limited and without remarkable differences between the two processed juices. Other research groups have previously demonstrated that some bioactive components (punicalin, punicalagin, ellagic acid and some anthocyanins) were cholinesterase inhibitors in the micromolar range [51], and thus their content can be important for this biological activity. Interestingly, the inhibitory activity against tyrosinase was more promising and statistically significant for PJ with respect to HP.

### 3.7. Eco-Toxicological Investigations

**Figure 4:**
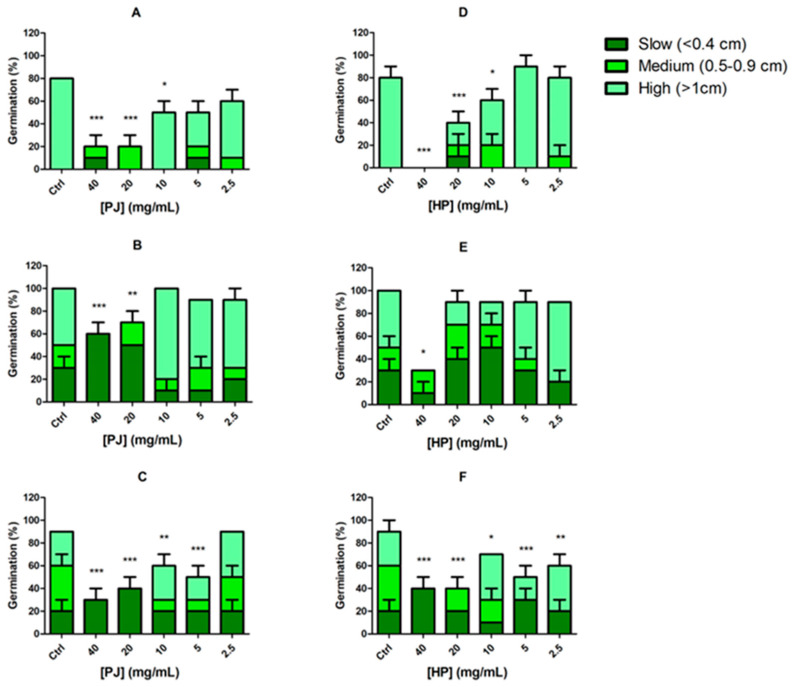
Germination and growth rate of *Cichorium intybus*, *Dichondra repens*, and *Diplotaxis tenuifolia* under different concentrations of *Punica granatum* PJ and HP. (**A**,**D**). Germination and growth rate of *Cichorium intybus* treated with PJ and HP, respectively. (**B**,**E**) Germination and growth rate of *Dichondra repens* treated with PJ and HP, respectively. (**C**,**F**) Germination and growth rate of *Diplotaxis tenuifolia* treated with PJ and HP, respectively. The bars represent germination percentages (%) at increasing juice concentrations (40, 20, 10, 5, and 2.5 mg/mL) compared to the control (Ctrl). Growth is categorized into three rates: slow (<0.4 cm, dark green), medium (0.5–0.9 cm, green), and high (>1 cm, light green). Data are presented as means ± SD. ANOVA, *p* < 0.0001; post hoc (Newman–Keuls test), * *p* < 0.05, ** *p* < 0.01, *** *p* < 0.001 vs. Ctrl (Control) group.

Allelopathy is a biological process by which plants secrete biochemicals into their environment, affecting the growth, survival, and reproduction of other plants. These allelochemicals can be present in various plant components [52]. In this study, we investigated the allelopathic effects of *Punica granatum* ultra-filtered (HP) and pasteurized (PJ) juices on the germination and growth of *Cichorium intybus*, *Dichondra repens*, and *Diplotaxis tenuifolia*. The study revealed a clear concentration-dependent allelopathic effect of both HP and PJ juices on the germination and growth of *C. intybus*, *D. repens*, and *D. tenuifolia*. This is consistent with the inhibitory effects induced by *P. granatum* peel water extract on seed germination and seedling growth of weeds [53]. Higher concentrations (40 and 20 mg/mL) of both extracts significantly inhibited germination and promoted slower growth across all three species (Figure 4A–F). In contrast, lower concentrations (10–2.5 mg/mL) enabled better germination rates and growth, approaching control levels and indicating reduced allelopathic effects at these concentrations with a consequent increase in biocompatibility. Species-specific responses were noted, with *C. intybus* and *D. tenuifolia* exhibiting more pronounced reductions in germination and growth at higher extract concentrations compared to *D. repens*. The dose-dependent phytotoxic effect induced by the extracts is consistent, albeit partially, with the content in phenolic compounds that are known to work as allelochemicals once released in the ground [54].

Both PJ and HP samples showed a concentration-dependent allelopathic effect, with higher concentrations (40 and 20 mg/mL) reducing germination and favoring slower growth rates. *Cichorium intybus* and *Diplotaxis tenuifolia* demonstrated greater sensitivity to juice treatments compared to *Dichondra repens.*

*Artemia salina*, also known as brine shrimp, is a zooplanktonic crustacean ubiquitous in saline aquatic environments ranging from lakes to oceans, and it is extensively utilized as a model system for the evaluation of acute toxicological responses. In particular, the brine shrimp lethality assay (BSLA) is widely used in preliminary screenings for bioactive compounds due to its simplicity, rapidity, reliability, and cost-efficiency [31].

This assay also demonstrates a high correlation of results with cytotoxic activity in higher organisms, in particular with the toxicity data of rodents and humans, and shows a good correlation with cytotoxicity tests, making these measurements suitable as preliminary results [55,56]. *Artemia* species have been used in testing the acute toxicity of toxic materials, such as heavy metals and pesticides [57], nanoparticles [58], bioactive molecules, natural extracts, and metal complexes [56].

The present study was conducted to test the toxicity limits of *P. granatum* HP and PJ. Toxicity limits were evaluated in terms of LC_50_ (lethality concentration) value. The determination of toxicity levels was based on LC_50_ values, using the standard toxicity indices established by Meyer and Clarkson. This classification helps in assessing the potential toxicity of various substances, including plant extracts, chemicals, and pharmaceuticals. According to Meyer’s classification, extracts are considered toxic if the LC_50_ is less than 1000 μg/mL and non-toxic if the LC_50_ is greater than 1000 μg/mL [55]. Clarkson’s classification categorizes substances as non-toxic for LC_50_ values above 1000 μg/mL, low toxic for LC_50_ values between 500 and 1000 μg/mL, medium toxic for LC_50_ values between 100 and 500 μg/mL, and highly toxic for LC_50_ values between 0 and 100 μg/mL [59].

**Figure 5:**
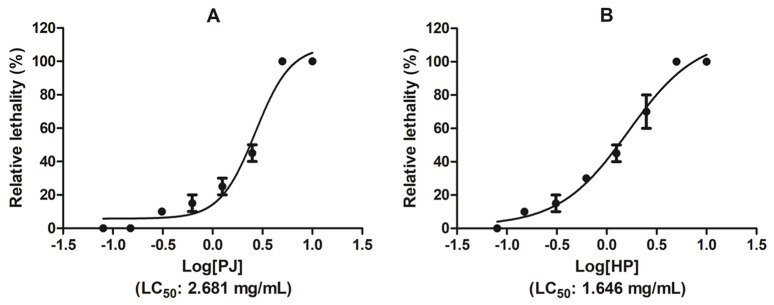
Dose–response relationship between *P. granatum* juices PJ (**A**) and HP (**B**) concentration and brine shrimp lethality. Toxicity levels were based on LC_50_ values.

Table 7 and Figure 4A,B show that *P. granatum* ultra-filtered (HP) and pasteurized (PJ) juices are both classified as non-toxic against *Artemia salina* nauplii (Figure 5), with LC_50_ values of 1.646 mg/mL and 2.681 mg/mL, respectively. These results are consistent with the non-significant toxicity by the methanol extract of *P. granatum* peel that showed an LC_50_ value of 1.42 mg/mL [60]. It is important to observe that the LC_50_ values calculated via brine shrimp assay and most of the results from the allelopathy assay pointed to a superimposable biocompatibility limit in different eukaryotic pluricellular organisms. However, considering the higher susceptibility of cell cultures, especially neuronal cells, to the cytotoxic effects of herbal extracts, a concentration range at least 5–10-fold lower compared with the present toxicity limits is recommended for further in vitro pharmacological studies [61].

## 4. Conclusions

The present study demonstrated that high-pressure processing (HP) better preserves anthocyanins compared to pasteurization (PJ), showing significantly higher anthocyanin content in the HP juice (approximately 130 mg/L vs. 76 mg/L). However, HP processing causes substantial degradation of ellagitannins, particularly punicalins and punicalagins, which were undetectable in HP juice but well represented in the pasteurized juice. Regarding ellagic acid, HP juice showed a higher concentration compared to PJ juice, suggesting that some degraded ellagitannins were converted into free ellagic acid. Pasteurization proved more effective in preserving vitamin C, likely due to enzymatic inactivation during thermal treatment. Metabolomic analyses using HPLC-DAD, DI-ESI-MS, and NMR confirmed the greater stability of anthocyanins in HP juice and their partial transformation into simpler compounds during processing. The total antioxidant capacity, measured through various assays, was higher in the pasteurized juice due to its higher concentration of complex polyphenolic compounds. Finally, ecotoxicity tests revealed that both juices are non-toxic toward *Artemia salina* and exhibit negligible concentration-dependent allelopathic effects on selected plant species. Overall, the study once again underlines that special attention must be paid to processing when it comes to achieving products selected by consumers for their healthy potential.

## Figures and Tables

**Table 1:** CIEL*a*b* parameters of PJ and HP.

	L*	a*	b*	C*_ab_	h_ab_			L*	a*	b*	C*_ab_	h_ab_
PJ	17.3	36.2	14.5	39.0	21.8		HP	8.3	23.8	4.7	24.3	11.1
PJ_1_	36.2	53.0	31.9	61.8	31.0		HP_1_	43.8	53.3	44.4	69.4	39.8
PJ_2_	37.1	53.4	33.4	63.0	32.0		HP_2_	45.4	52.1	44.6	68.6	40.6
PJ_3_	24.0	49.1	29.4	57.2	30.9		HP_3_	15	44.2	25.7	51.1	30.2

Not shown RSD values are within 2–3%.

**Table 2:** Quali-quantitative data from HPLC-DAD analyses.

	PJ (Mean Value ± SD)	HP (Mean Value ± SD)
Vitamin C	284.0 ± 39.1 ^a^	185.2 ± 27.0 ^a^
Punicalins	405.5 ± 20.3	tr *
Punicalagins	94.1 ± 4.71	nd **
Ellagic acid derivative	36.6 ± 1.83 ^b^	28.8 ± 1.44 ^b^
Ellagic acid glucoside	24.5 ± 1.22 ^c^	14.4 ± 0.72 ^c^
Ellagic acid	12.0 ± 0.60 ^c^	33.8 ± 1.69 ^c^
Del-3,5-diglucoside	17.8 ± 0.89 ^c^	32.9 ± 1.65 ^c^
Cya-3,5-diglucoside	19.9 ± 1.12 ^c^	61.8 ± 3.09 ^c^
Del-3-glucoside	14.3 ± 0.71	13.5 ± 0.68
Cya-3-glucoside	17.9 ± 0.90 ^a^	21.7 ± 1.08 ^a^
∑ of anthocyanins	75.6 ± 3.78 ^c^	129.7 ± 6.48 ^c^

Vitamin C is expressed in mg/Kg juice ± SD. Ellagitannins and anthocyanins expressed in mg/L juice ± SD; * traces; ** undetectable. Different letters indicate significant differences between the tested samples: ^a^ *p* < 0.05; ^b^ *p* < 0.01; ^c^ *p* < 0.001.

**Table 3:** Metabolite profile traced by DI-ESI-MS analysis.

**Positive Ion Mode**			**Intensity %**	
**Compound**	***m*** **/*z***	**MS-MS Fragments**	**PJ**	**HP**
Pel-3-glucoside	433	271	7 ± 0.06	6 ± 0.05
Cya-3-glucoside	449	287	40 ± 0.15	39 ± 0.12
Del-3-glucoside	465	303	13 ± 0.09	10 ± 0.10
Cya-3-5-pentoside-hexoside	581	449, 419, 287	3 ± 0.02	3 ± 0.02
Pel-3,5-diglucoside	595	433	8 ± 0.04	10 ± 0.05
Cya-3,5-diglucoside	611	449, 287	24 ± 0.12	29 ± 0.11
Del-3-,5-diglucoside	627	465, 303	5 ± 0.03	3 ± 0.04
**Negative ion mode**				
**Compound**	***m*** **/*z***	**MS-MS fragments**		
Citric acid	191	173, 111		
Citric acid derivative		67		

**Table 4:** ^1^H NMR signals and quantitative results of detected metabolites in juice samples *.

Amino Acids and Derivatives			
Metabolite	^1^H ppm, Multiplicity *J* [Hz]	PJ mg/mL ± SD	HP mg/mL ± SD
Alanine	1.48, d [7.2]	0.28 ± 0.06	0.13 ± 0.02
Asparagine	2.80, dd [7.3; 16.9]	1.61 ± 0.04 ^a^	0.30 ± 0.02
Aspartate	2.85, dd [17.5; 3.9]	0.39 ± 0.04 ^a^	0.13 ± 0.03
GABA	1.93, m	0.70 ± 0.12	0.53 ± 0.08
Glutamine	2.15, m	1.25 ± 0.22	0.82 ± 0.08
Isoleucine	1.01, d [7.0]	0.03 ± 0.001 ^a^	0.01 ± 0.001
Leucine	0.95, t [6.2]	0.03 ± 0.001 ^a^	0.01 ± 0.001
Phenylalanine	7.35, m	0.27 ± 0.05 ^a^	0.10 ± 0.01
Threonine	1.34, d [6.6]	0.05 ± 0.01	0.02 ± 0.01
Tyrosine	6.91, d [8.5]	0.07 ± 0.01	0.03 ± 0.01
Valine	1.05, d [7.0]	0.09 ± 0.02	0.03 ± 0.01
**Sugars and polyols**			
Fructose	4.13, m	38.88 ± 2.57 ^a^	16.8 ± 1.55
Galactose	4.60, d [8.0]	0.12 ± 0.08	0.08 ± 0.02
Glucose	4.66, d [8.0]	128.39 ± 16.19	84.4 ± 13.26
Maltose	5.52, d [3.9]	0.52 ± 0.04	0.25 ± 0.04
Sucrose	5.40, d [3.8]	0.66 ± 0.13	0.33 ± 0.05
Myo-inositol	3.30, t [9.3]	1.77 ± 0.17 ^a^	0.68 ± 0.12
**Organic acids**			
Citric acid	2.80, d [15.9]	46.80 ± 4.63	19.6 ± 4.16
Formic acid	8.46, s	0.003 ± 0.001	0.8 ± 0.1 (µg/mL)
Fumaric acid	6.62, s	0.005 ± 0.001	0.004 ± 0.001
Gallic acid	7.08, s	0.17 ± 0.01	0.28 ± 0.02
**Other metabolites**			
Ethanol	1.24, t [7.06]	0.19 ± 0.04	0.12 ± 0.02
Uridine	7.87, d [8.1]	0.01 ± 0.001	0.01 ± 0.001
Trigonelline	9.11, s	0.07 ± 0.02	0.05 ± 0.01

* Expressed as mg/mL of fresh juice ± SD. ^a^ significative difference respect to HP sample, (*p* < 0.0001).

**Table 5:** Total phenolic and flavonoid content and antioxidant properties * of the pomegranate samples.

Sample	TPC (mg GAE/g)	TFC (mg RE/g)	DPPH (mg TE/g)	ABTS (mg TE/g)	CUPRAC (mg TE/g)	FRAP (mg TE/)	Chelating (mg EDTAE/g)	PBD (mmol TE/g)
PJ	15.9 ± 0.16 ^a^	5.0 ± 0.20 ^a^	55.1 ± 0.74 ^a^	91.8 ± 0.78 ^a^	74.5 ± 1.07 ^a^	75.3 ± 1.04 ^a^	1.04 ± 0.03 ^a^	1.54 ± 0.01 ^a^
HP	11.5 ± 1.53 ^b^	1.9 ± 0.48 ^b^	35.1 ± 0.74 ^b^	51.5 ± 1.71 ^b^	47.3 ± 0.81 ^b^	48.2 ± 1.13 ^b^	0.40 ± 0.01 ^b^	1.26 ± 0.07 ^b^

* Values are reported as mean ± SD of three parallel measurements. TPC: Total Phenolic Content; TFC: Total Flavonoid Content. GAE: gallic acid equivalent; RE: rutin equivalent. PBD: phosphomolybdenum; TE: Trolox equivalent; EDTAE: EDTA equivalent. Different letters indicate significant differences between the tested samples (*p* < 0.05).

**Table 6:** Enzyme inhibitory properties * of the pomegranate samples.

Juice	AChE (mg ALAE/g)	BChE (mg GALAE/g)	Tyrosinase (mg KAE/g)	Amylase (mmol ACAE/g)	Glucosidase (mmol ACAE/g)
PJ	2.52 ± 0.03 ^a^	1.31 ± 0.25 ^a^	32.44 ± 0.87 ^a^	0.03 ± 0.01 ^a^	2.74 ± 0.04 ^a^
HP	2.48 ± 0.03 ^a^	1.52 ± 0.19 ^a^	24.66 ± 1.78 ^b^	0.04 ± 0.01 ^a^	2.35 ± 0.10 ^b^

* Values are reported as mean ± SD of three parallel measurements. GALAE: galantamine equivalent; ACAE: acarbose equivalent; KAE: kojic acid equivalent. Different letters indicate significant differences between the samples (*p* < 0.05).

**Table 7:** Brine shrimp lethality *.

Juice	Concentration Range [mg/mL]	LC_50_ (mg/mL)	95% Confidence Interval	R^2^	Toxicity Class	
					Meyer’s Classification	Clarkson’s Classification
PJ	[0.078–10]	2.681	2.058–3.492	0.967	non-toxic	non-toxic
HP	[0.078–10]	1.646	1.066–2.541	0.975	non-toxic	non-toxic

* Tested samples in a concentration range between 0.078 and 10 mg/mL.

## Data Availability

The original contributions presented in this study are included in the article/Supplementary Materials. Further inquiries can be directed to the corresponding author.

## Supplementary Materials

The following supporting information can be downloaded at: https://www.mdpi.com/article/10.3390/foods14020315/s1, Equation S1: Calculated color difference; Table S1: Standard calibration curves; Figure S1: Standard chromatogram registered at 360 nm; Figure S2: Standard chromatogram registered at 520 nm.
