# Supplementary material for: Pomegranate Juices: Analytical and Bio-Toxicological Comparison of Pasteurization and High-Pressure Processing in the Development of Healthy Products
Source: Foods. 2025 Jan 18;14(2):315. doi: 10.3390/foods14020315 (PMC11765445; doi:10.3390/foods14020315)
Supplement: Supplementary file 1 [file foods-14-00315-s001.zip › foods-3429338-supplementary.pdf]

Pomegranate Juices: Analytical and Bio-Toxicological Comparison of Pasteurization and High-Pressure Processing in the Development of Healthy Products

Francesco Cairone [1], Stefania Cesa [1,*], Irene Arpante [1], Simonetta Cristina Di Simone [2], Alejandro Han Mendez [1], Claudio Ferrante [2], Luigi Menghini [2], Antonello Filippi [1], Caterina Fraschetti [1], Gokhan Zengin [3], Simone Carradori [2], Marialucia Gallorini [2], Luisa Mannina [1] and Mattia Spano [1]

[1] Department of Chemistry and Technologies of Drug, "Sapienza" University of Rome, 00185 Rome, Italy; francesco.cairone@uniroma1.it (F.C.); irene.arpante@uniroma1.it (I.A.); alejandrohan.mendez@uniroma1.it (A.H.M.); antonello.filippi@uniroma1.it (A.F.); caterina.fraschetti@uniroma1.it (C.F.); luisa.mannina@uniroma1.it (L.M.); mattia.spano@uniroma1.it (M.S.)
[2] Department of Pharmacy, University "G. d'Annunzio" of Chieti-Pescara, 66100 Chieti, Italy; simonetta.disimone@unich.it (S.C.D.S.); claudio.ferrante@unich.it (C.F.); luigi.menghini@unich.it (L.M.); simone.carradori@unich.it (S.C.); marialucia.gallorini@unich.it (M.G.)
[3] Department of Biology, Science Faculty, Selcuk University, Konya 42130, Turkey; gokhanzengin@selcuk.edu.tr
* Correspondence: stefania.cesa@uniroma1.it

Equation S1. Calculated color difference

$$\Delta E=\sqrt{(L_2 - L_1)^2 + (a_2 - a_1)^2 + (b_2 - b_1)^2}$$

Table S1. Standards calibration curves

Name	Wavelength (nm)	Retention Time (min)	Calibration Curve	R^2
Punicalin α+β	360	5.0 - 5.2	*y*= 8.43x + 83.61	0.9977
Punicalagin α+β		6.9; 9.7	*y*= 4.00x + 2.88	0.9996
Ellagic Acid		37.1	*y*= 11.60x – 38.34	0.9995
Cyanidin-3-glucoside	520	24.2	y = 16.61x - 77.14	0.9994

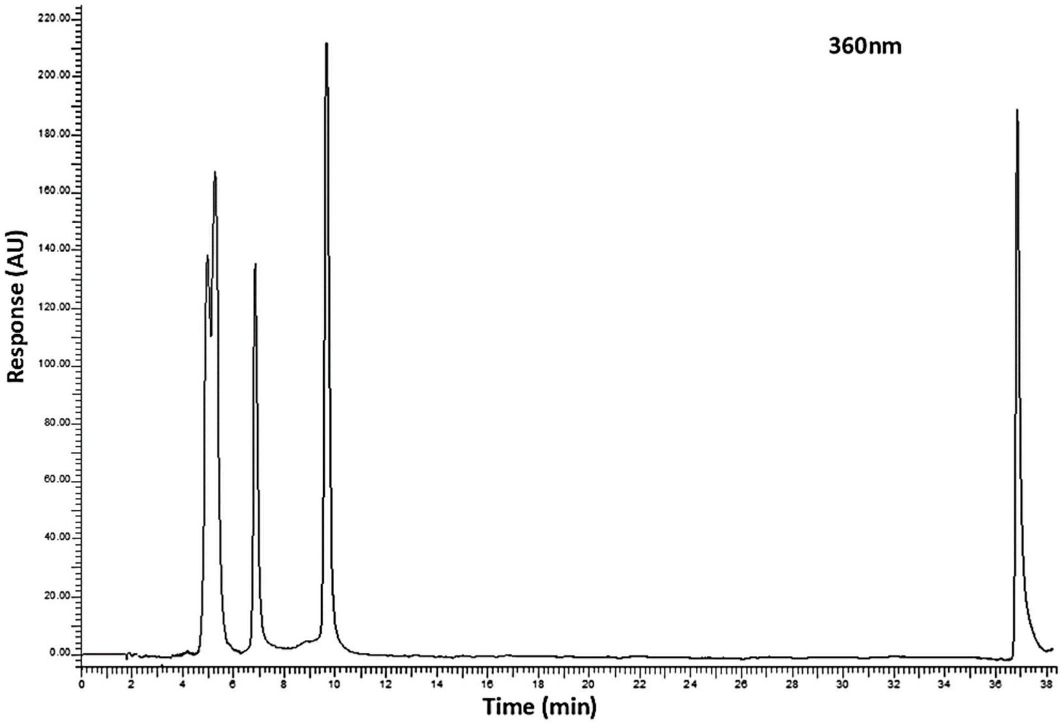

Figure S1. Standards chromatogram registered at 360 nm.

Punicalin (α+β), R_T = 5.0-5.2; Punicalagin (α and β), RT = 6.9 and 9.7; Ellagic acid, RT = 37.1.

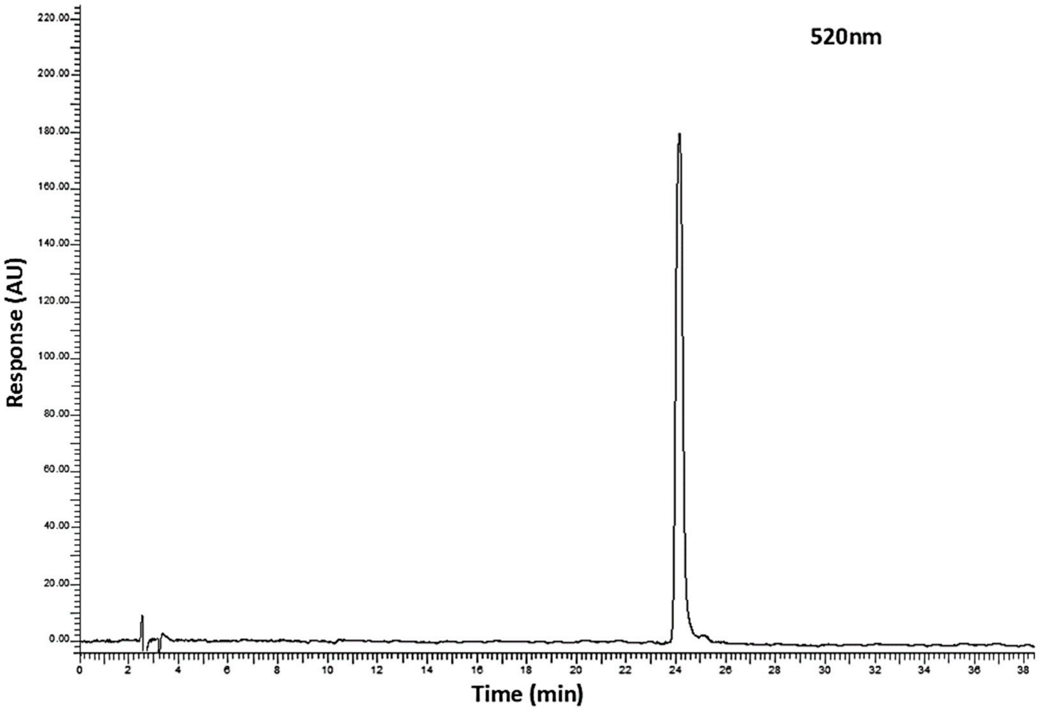

Figure S2. Standard chromatogram registered at 520 nm.

Cyanidin-3-glucoside, RT = 24.2.
